# The electronic self report assessment and intervention for cancer: promoting patient verbal reporting of symptom and quality of life issues in a randomized controlled trial

**DOI:** 10.1186/1471-2407-14-513

**Published:** 2014-07-12

**Authors:** Donna L Berry, Fangxin Hong, Barbara Halpenny, Anne Partridge, Erica Fox, Jesse R Fann, Seth Wolpin, William B Lober, Nigel Bush, Upendra Parvathaneni, Dagmar Amtmann, Rosemary Ford

**Affiliations:** 1 Department of Biobehavioral Nursing and Health Systems, University of Washington, Box 357366, Seattle, WA 98195-7366, USA; 2 Phyllis F. Cantor Center, Dana-Farber Cancer Institute, 450 Brookline Ave, LW 518, Boston, MA 02215, USA; 3 Biostatistics & Computational Biology, Dana-Farber Cancer Institute, 450 Brookline Ave, Boston, MA 02115, USA; 4 Dana-Farber Cancer Institute, Department of Medicine, Harvard Medical School, 450 Brookline Ave, Boston, MA 02215, USA; 5 Department of Psychiatry, University of Washington Medical Center, Seattle, WA 98195, USA; 6 Seattle Cancer Care Alliance, 825 Eastlake Ave E, Seattle, WA 98109, USA; 7 U.S. Department of Defense, Joint Base Lewis-McChord, National Center for Telehealth and Technology, Tacoma, Washington, USA; 8 Radiation Oncology, University of Washington Medical Center, Seattle, WA 98195, USA; 9 Department of Rehabilitation Medicine, University of Washington Seattle, Box 354237, Seattle, WA 98195-4237, USA

**Keywords:** Patient-provider communication, Cancer, Symptoms, Coaching, Internet

## Abstract

**Background:**

The electronic self report assessment - cancer (ESRA-C), has been shown to reduce symptom distress during cancer therapy The purpose of this analysis was to evaluate aspects of how the ESRA-C intervention may have resulted in lower symptom distress (SD).

**Methods:**

Patients at two cancer centers were randomized to ESRA-C assessment only (control) or the Web-based ESRA-C intervention delivered to patients’ homes or to a tablet in clinic. The intervention allowed patients to self-monitor symptom and quality of life (SxQOL) between visits, receive self-care education and coaching to report SxQOL to clinicians. Summaries of assessments were delivered to clinicians in both groups. Audio-recordings of clinic visits made 6 weeks after treatment initiation were coded for discussions of 26 SxQOL issues, focusing on patients’/caregivers’ coached verbal reports of SxQOL severity, pattern, alleviating/aggravating factors and requests for help. Among issues identified as problematic, two measures were defined for each patient: the percent SxQOL reported that included a coached statement, and an index of verbalized coached statements per SxQOL. The Wilcoxon rank test was used to compare measures between groups. Clinician responses to problematic SxQOL were compared. A mediation analysis was conducted, exploring the effect of verbal reports on SD outcomes.

**Results:**

517 (256 intervention) clinic visits were audio-recorded. General discussion of problematic SxQOL was similar in both groups. Control group patients reported a median 75% of problematic SxQOL using any specific coached statement compared to a median 85% in the intervention group (p = .0009). The median report index of coached statements was 0.25 for the control group and 0.31 for the intervention group (p = 0.008). Fatigue, pain and physical function issues were reported significantly more often in the intervention group (all p < .05). Clinicians' verbalized responses did not differ between groups. Patients' verbal reports did not mediate final SD outcomes (p = .41).

**Conclusions:**

Adding electronically-delivered, self-care instructions and communication coaching to ESRA-C promoted specific patient descriptions of problematic SxQOL issues compared with ESRA-C assessment alone. However, clinician verbal responses were no different and subsequent symptom distress group differences were not mediated by the patients' reports.

**Trial registration:**

NCT00852852; 26 Feb 2009

## Background

Patient-clinician communication has been evaluated and found lacking with regard to clinician assessment of patient experiences, notably symptoms and quality-of-life issues (SxQOL) [1-3], and verbal patient reports of SxQOL [4]. Barriers to communication in the oncology setting have been identified and include 1) clinician-oriented verbal behaviors: use of close-ended (versus open-ended) queries and interruptions of patient symptom descriptions [5,6], changing the subject after a patient verbally reports an SxQOL; [7] 2) clinician beliefs that quality of life issues are other clinicians' responsibility [8], 3) patient-oriented issues: reluctance to verbalize problems [9], recall of SxQOL experiences in between visits [10], and 4) time limitations during the visit [11]. When clinicians are unaware of SxQOL, particularly treatment-related toxicities, there is danger of higher morbidity and even mortality related to unintentional over-dosing [12,13]. Interventions to improve patient-clinician communication have been tested with modest, but positive, results [9,14-16].

In the first electronic self report assessment for cancer (ESRA-C) randomized clinical trial [17], we demonstrated the feasibility, acceptability and efficacy of computerized SxQOL screening at a large comprehensive cancer center in Seattle, significantly increasing the frequency of patient/clinician communication about problematic issues as measured in audio-recorded clinic visits. Yet, we found that even when clinicians received summaries of patient-reported SxQOL, the most frequently addressed issues were those either regulated by certification bodies (e.g., pain) or likely to be affected by supportive care medications previously ordered by the clinician (e.g., nausea with anti-emetics). High distress SxQOL reported by patients on the ESRA-C measure were often left unaddressed by clinicians [6,7]. A second randomized trial (ESRA-C II) in which the clinician summary intervention was delivered for all participant clinic visits, tested a new intervention that offered SxQOL tracking, tailored education and communication coaching directly to patients recruited from two comprehensive cancer centers. The results from ESRA-C II indicated significantly lower symptom distress over the course of therapy with the intervention [18]. Because ESRA-C was a multi-component intervention, we wanted to understand more about the impact of the communication coaching on the verbal behaviors of patients during the face-to-face visit. The purpose of this analysis was to compare verbal reports of SxQOL between the study groups with regard to: 1) reported severity, pattern, alleviating/aggravating factors and requests for help for the full set of 26 ESRA-C SxQOL issues; and 2) reports of individual SxQOL issues within the full set, plus 3) to determine whether any observed differences would account for differences in symptom distress.

## Methods

**Figure 1:**
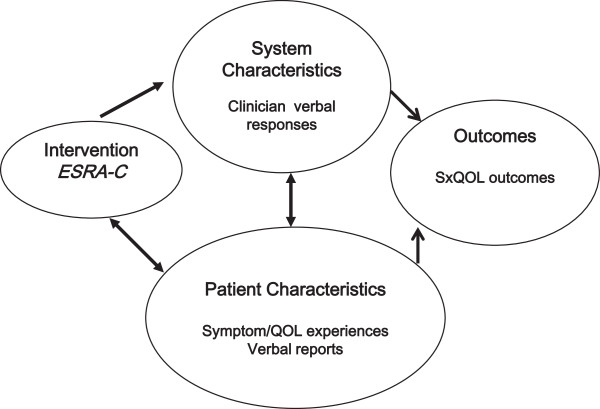
Health outcomes model adapted to these analyses.

This analysis is one component of a program of research founded on the Quality Health Outcomes Model, a framework proposed by Mitchell and colleagues [19] to illustrate that patient outcomes are rarely explained only by specific interventions but also by health care system/provider factors and patient-specific factors. Patients' verbal behaviors can be placed in the model (Figure 1) as a patient-specific factor that may mediate, along with setting factors such as clinician verbal behaviors, the impact of the ESRA-C intervention on symptom distress.

### Design, sample, intervention

**Figure 2:**
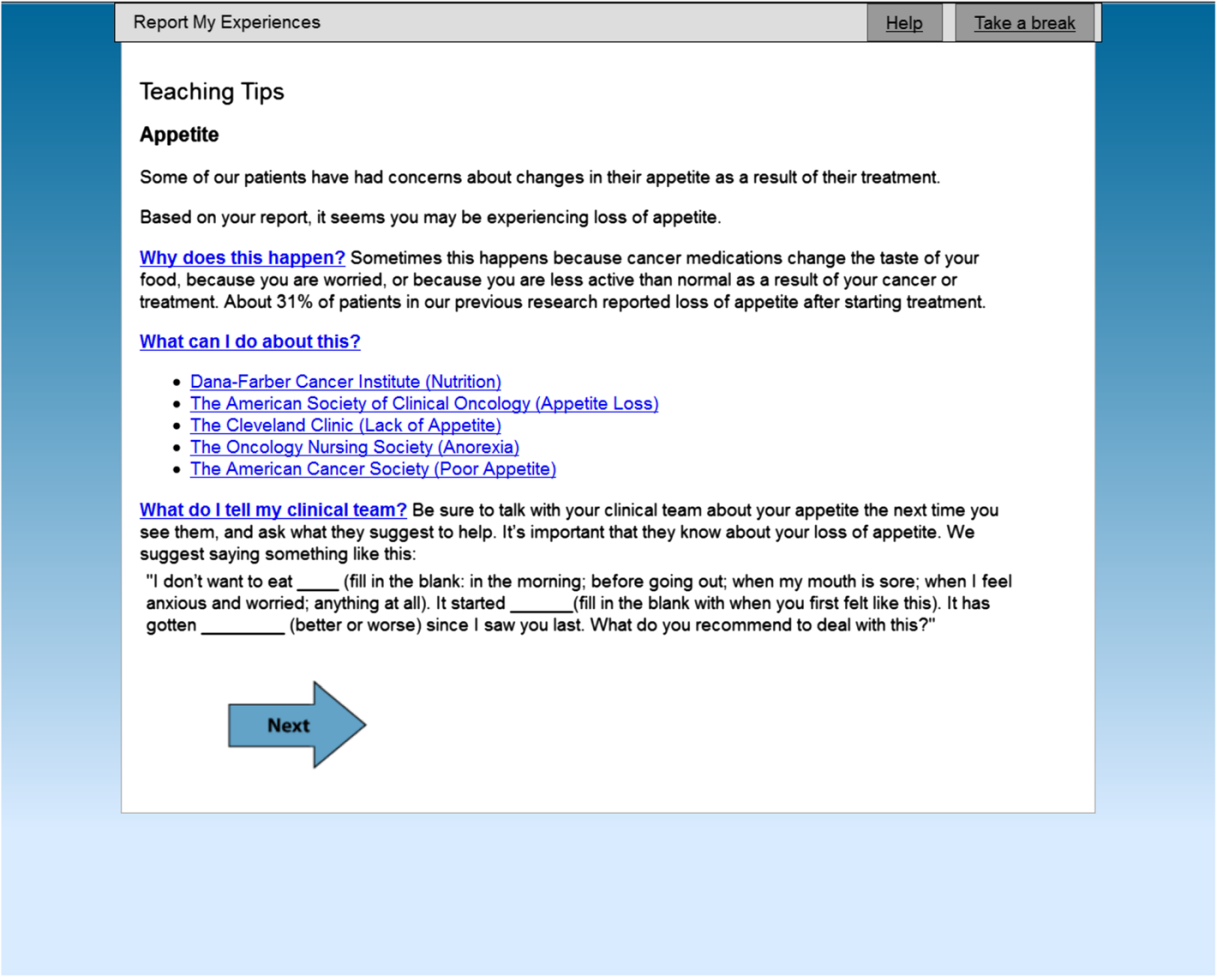
Exemplar of online coaching regarding self-care and communication.

The ESRA-C II trial was a randomized trial conducted at two comprehensive cancer centers. The study was approved by both the Dana-Farber/Harvard Cancer Center and the Fred Hutchinson Cancer Institute Institutional Review Boards. Participants were patients with various cancers at a range of stages. The details of the trial were reported elsewhere [18]. In brief, adult patients who provided written informed consent to participate and were about to start a new medical or radiation anti-cancer therapy were randomized to receive usual education about SxQOL topics or usual education plus tailored self-care instruction for moderate-to-severe reported SxQOL issues. In both arms, patients reported SxQOL using the ESRA-C and clinicians received summaries of patient-reported SxQOL prior to treatment (T1) and again within 24 hours prior to a face-to-face clinic visit (T2). Cancer symptomatology was measured primarily with the Symptom Distress Scale-15 (SDS-15), adapted from the 13-item, legacy instrument developed by McCorkle and Young [20] and validated in many subsequent studies and languages [21]. The SDS-15 offers patients with cancer the opportunity to report most of the common symptoms and side effects of therapy in an easy-to-understand format. Patients in the intervention group could access the ESRA-C program from home or in clinic on a touch-screen computer at any time throughout the trial to electronically track SxQOL and view self-care instruction.The patient instruction in the intervention arm included on-screen, tailored coaching on how to better communicate each troublesome SxQOL issue to the clinician, and specifically to remind and encourage the patient to describe the severity, pattern, and alleviating/aggravating factors related to the issue, and to ask for assistance in managing the issue. Figure 2 depicts an exemplar of the communication coaching text. These coaching instructions were delivered immediately before each on-study clinic visit for the 14.5% (109/752) of the sample without remote access to the ESRA-C program and within 24 hours of the visits for those patients with remote access at home (85.5%).

About six weeks after study enrollment and treatment initiation (T2), a regularly scheduled clinic visit between the participant and clinician was audio-recorded. All recordings were cleaned of potential identifiers. Research team members listened to the recordings using Sound Forge Audio Studio software version 9 (Sony Creative Software, Middleton, WI) and coded the following for each of 26 SxQOL issues (including a field for free text entry of SxQOL not specifically assessed): a) who initiated; b) whether the issue was discussed and defined as problematic; and c) which, if any, of the four coached descriptions/requests (severity, pattern, alleviation/aggravation and request for help) the patient and/or caregiver made without clinician prompting. We defined the SxQOL issue problematic when it was discussed as current at any degree of severity.

Members of the study team were trained to code recordings and completed eight practice cases with feedback to achieve proficiency prior to initiating coding. The team met to review and discuss coding monthly over fourteen months. Coders were blinded to group assignment unless it was disclosed during the course of the recorded clinic visit. Cases were assigned for coding randomly. Twelve percent of cases were randomly selected to be double-coded for reliability; percent agreement was calculated in which every matched code was an agreement (for example, both coders identify an utterance as a description of the pattern of a particular symptom), and every unmatched code was a disagreement (for example, one coder identified the patient and another the family member as the initiator of a particular symptom discussion). When there were disagreements, the coders reviewed codes together and determined the final codes used for analysis.

### Analytic methods

Descriptive statistics were used to summarize baseline sample characteristics and numbers of SxQOL issues identified as problems in the audio-recorded visits. First, the percentage of problematic issues initiated by the patient, family or clinician was calculated. Among problematic SxQOL issues, two measures were defined for analysis across all patients: 1) the percentage of problematic issues about which the patient or caregiver verbally reported at least one coached statement without clinician prompting; and 2) a report index of how many coached statements were made by the patient during the visit), regarding problematic SxQOL issues (for example, if a patient reported only severity for fatigue, the measure would be 1/4 = .25).

The range of both measures was from 0 to 1. The Wilcoxon rank test was used to compare the measures between study groups. Covariates previously identified as influencing symptom distress in the primary outcome analysis (age, clinical service, working status and baseline SDS-15 score) were adjusted in multivariable analysis to improve the precision of estimating the intervention effect on the two scores. Two-way interactions between study group and other covariates were tested and none were found significant. In addition, for each individual SxQOL, the percentage of problematic SxQOL reported as coached was compared between groups with a Fisher’s Exact test.

In order to assess whether patient verbal reports mediated the outcome of the primary analysis [18], reduced symptom distress, we conducted a mediation analysis using a causal step approach [22]. Specifically, for the analytic sample (participants with audio recordings), the difference in SDS-15 from baseline to study end was calculated. Then three regression analyses were performed: 1) SDS-15 difference on study group, adjusting for baseline SDS-15 score; 2) patient verbal report measure on study group; and 3) SDS-15 difference on study group and patient verbal report measure, adjusting for baseline SDS-15 score. The mediation would be established if all of the following were observed: significant relationships in regression 1 and 2; significant relationship of patient verbal report measure with SDS-15 difference in regression 3; and a smaller coefficient of the study group in regression 3 compared with regression 1. We tested the mediation effect of each patient verbal report measure separately. Lastly, the percentage of problematic SxQOL issues for which clinicians verbalized a treatment or referral was calculated for each patient and compared between two study group with the Wilcoxon rank test. For all tests, a two-sided p-value of 0.05 was considered statistically significant and 0.1 was considered to indicate a trend.

## Results

**Figure 3:**
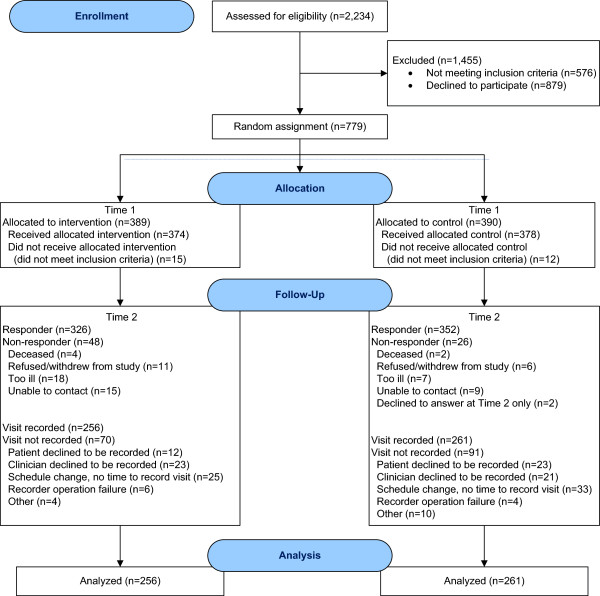
Analytic sample of 517 audio recorded clinic visits.

Among 752 eligible patients, 517 clinic visits were audio-recorded and coded for analysis (Figure 3). In one recording, the patient referenced teaching material in the online intervention, effectively un-blinding the coder to group assignment. Sixty-two recordings were double-coded, with a mean percent agreement of 86.7 (median 88.0; range 61.0-99.5). Of the recordings available for analysis, 261 were from the control group patients and 256 from the intervention group. Baseline participant characteristics are presented by study group in Table 1. Patients with audio-recordings were younger in the intervention group than in the control group (p < .0001). Out of 517 patients, 27 (13 control and 14 intervention) did not discuss any problematic SxQOL issue during the clinic visits. There was no significant difference (p = 0.41) between study groups in number of problematic SxQOL issues discussed at all during clinic visits, with a median of 4 issues discussed by control group patients, and 3 by patients in the intervention condition. Patients initiated general discussion of an average 56% of problematic SxQOL issues in the control group and 55% in the intervention group (p = 0.97). Family members initiated 4% of the problematic SxQOL issues in the control group and 5% in the intervention group (p = 0.35).

**Table 1:** Baseline patient characteristics for those with an audio-recorded visit (N = 517)

	**Study group**			
	**Control**		**Treatment**	
	**n = 261**		**n = 256**	
	**n**	**(%)**	**n**	**(%)**
**Age**				
< 50	56	21.5	96	37.5
≥ 50	205	78.5	160	62.5
Median (range)	59	(22–87)	55	(22–86)
**Gender**				
Male	142	54.4	123	48.0
Female	119	45.6	133	52.0
**Clinical Service**				
Medical Oncology	144	55.2	151	59.0
Radiation Oncology	87	33.3	86	33.6
HSCT	30	11.5	19	7.4
**Working Status**				
Missing	33	12.6	16	6.3
Not Working	72	27.6	81	31.6
Working full/part time	156	59.8	159	62.1
**Cancer Type**				
Bladder	8	3.1	5	2.0
Breast	70	26.8	81	31.6
Colorectal	21	8.0	28	10.9
Gastrointestinal, not colorectal	47	18	47	18.3
Head and Neck	15	5.7	12	4.7
Leukemia/lymphoma/myeloma	41	15.7	30	11.8
Prostate	48	18.4	49	19.1
Other	21	8	16	6.3
Missing	8	3.1	5	1.9
**Stage**				
0	8	3.1	2	0.8
1	44	16.9	39	15.2
2	60	23.0	67	26.2
3	38	14.6	56	21.9
4	73	28.0	65	25.4
N/A	3	1.1	0	0
Missing	35	13.4	27	10.5
**SDS-15 (T2) mean (SD)**	27.0 (8.12)		26.6 (7.73)	

SD, standard deviation.

The percentage of problematic SxQOL issues which patients or caregivers reported using any specific coached statement during the clinic visit, was significantly higher (p = 0.002) in the intervention group than that in the control: a median of 85% of problematic SxQOL were reported as coached in the intervention group versus 75% in the control (Table 2). After adjusting for covariates, group remained significantly associated (p = 0.0009); intervention group patients had an approximate 9% higher rate of describing problems with a coached statement (Table 3).

**Table 2:** Problematic SxQOL verbally reported with any coached statement(s) and index of coached statements per SxQOL (N = 517)

	**% reported by patients**		**Index of coached statements reported by patients**	
	**Median**	**Mean (SD)**	**Median**	**Mean (SD)**
	**(Q1, Q3)**		**(Q1, Q3)**	
Control (n = 261)	0.75	0.68 (0.32)	0.25	0.29 (0.18)
	(0.50, 1.00)		(0.17, 0.40)	
Treatment (n = 256)	0.85	0.77 (0.27)	0.31	0.33 (0.17)
	(0.60, 1.00)		(0.21, 0.43)	

Q1 = lower quartile (25^th^ percentile).

Q3 = upper quartile (75^th^ percentile).

**Table 3:** Multivariable regression analysis of percentage of problematic SxQOL which patients reported using any coached statement, and of index of coached statements (N = 517)

	**% SxQOL reported by patients**			**Index of coached statements reported by patients**		
	**Est.**	**95% CI**	**P-value**	**Est.**	**95% CI**	**P-value**
**Study Group**	0.090	0.033 to 0.15	0.002	0.037	0.003 to 0.071	0.03
(Intervention vs. control)						
**Age**	−0.0000	−0.0024 to 0.0024	1.0	−0.0005	−0.0020 0.0009	0.5
**Service**			0.10			0.1
HSCT vs. RadOnc	0.10	−0.0015 to 0.20	0.05	0.042	−0.018 to 0.10	0.2
MedOnc vs. RadOnc	0.055	−0.0094 to 0.12	0.09	0.039	0.0006 to 0.077	0.05
**Work Status**	−0.0001	−0.064 to 0.063	1.0	0.0018	−0.036 to 0.040	0.9
(Not working vs. working full/part time)						
**Baseline SDS15**	0.0017	−0.0023 to 0.0056	0.4	0.0014	−0.0010 to 0.0037	0.3

**Figure 4:**
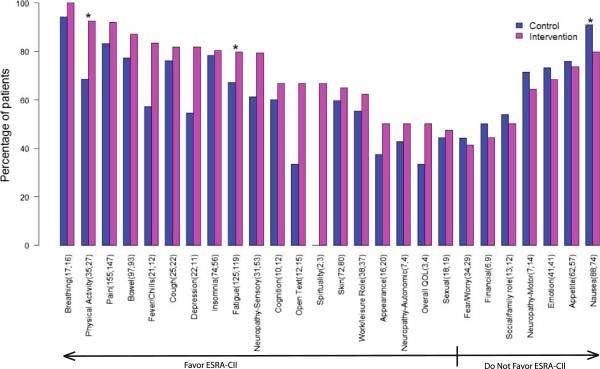
**Percentage of problematic SxQOL issues reported as coached, by study group.** For each SxQOL issue, the number of patients' visits (n control, n intervention) in which the issue was defined as a problem is shown and the percentage of visits in which the patient or caregiver made unprompted reports of severity, pattern, or alleviating/aggravating factors, or requested help for the SxQOL issue. (*) denotes a p-value of ≤ .05 for the difference between study groups in reporting percentage.

The report index of coached statements was significantly higher (p = 0.01) in the intervention group than in the control group, with medians of 0.31 and 0.25, respectively (Table 2). In other words, a patient with four discussed problematic issues in the intervention group gave one more coached, specific statement than a similar patient in the control. In the multivariable model adjusting for potential covariates (Table 3), study group was the only factor significantly associated (p = 0.03) with report index scores. Patients in the intervention group had an average 0.036 higher report index than those in the control group.Figure 4 displays the percentage of participants reporting any coached statement for each problematic SxQOL. The most frequently described issues in both groups were those related to symptoms versus quality of life domains. The percentage in the intervention group was significantly higher for fatigue (p = 0.03), pain (p = 0.02) and physical function (p = 0.02), and trended higher for bowel (p = 0.08), sensory neuropathy (p = 0.07), and SxQOL issues reported by patients in free text entry (p = 0.09). However, the percentage of specific, coached descriptions of nausea was significantly lower in the intervention group (p = 0.04).

Of 517 patients with audio data, 445 had SDS-15 scores at baseline and the study endpoint, and thus were included in the mediation analysis. Significant relationships were confirmed between study groups and SDS-15 difference (p = 0.05) in the first regression, and study group and patient verbal report measures in the second regressions (p = 0.0005 for percent of problematic SxQOL issues reported using any coached statement, and p = 0.01 for the report index). However, when the SDS-15 difference was regressed on study group and patient verbal report measures in the final pair of analyses, neither of the two verbal report measures was significant (p = 0.26 for percent of problematic SxQOL reported using any coached statement, p = 0.41 for the report index ). The results suggest that patients' specific verbalization of coached statements did not mediate the impact of the ESRA-C intervention on symptom distress. During the recorded clinic visits, clinicians verbalized treatment or a referral for a median of 48% of problematic SxQOL issues in the control group and a median of 50% in the intervention group (p = 0.15).

## Discussion

The patients in the ESRA-C II randomized trial who received an educational coaching intervention to aid verbal report of problematic SxQOL applied the reporting framework as coached (severity, pattern, aggravating/alleviating factors and help request), reporting these specific details without prompting, significantly more often than control group patients. When examining individual SxQOL issues, we found that reports for the majority of individual SxQOL issues were more frequent in the intervention group. Even though our study was not powered to compare individual SxQOL issues, we found that fatigue, pain and physical function were reported significantly more often by the intervention group. Fatigue is known to be the most common cancer symptom [23], pain also has a high incidence, and physical functioning is impacted by both. Interestingly, specifics of problematic nausea were reported significantly less often in the intervention group. This may reflect that intervention patients were intent on reporting other SxQOL issues perceived to be more important or more difficult to manage, and consequently, nausea was not one of the priorities.

Findings from our previous randomized trial with ESRA-C [17] and from Velikova et al. [16] established the significant and positive effect of a clinician summary on verbal discussions of SxQOL within a face-to-face clinic visit, yet neither trial significantly increased patient verbal self-reports. Instructing the participant to verbally report the same information reported on the quantitative SxQOL questionnaires was not included in these earlier trials. Of all the trials conducted with SxQOL outcomes, very few have utilized a direct measure of patient-provider communication [24]. Wilkie and colleagues [25] randomized 151 patients with lung cancer and found significantly more unsolicited reports of pain intensity in audio-recordings of on-treatment clinic visits after an intervention consisting of a coaching videotape plus personal reinforcement. Street et al. [26] reported that, in 148 patients with cancer randomized to an educational communication coaching intervention, higher baseline pain and several demographic variables predicted more pain-specific active participation in the clinic visit conversation. Taking all of these results together, coaching patients with cancer to engage in conversations with specialists monitoring the treatment course shows promise as an adjunct to providing clinicians with quantitative information from SxQOL questionnaires. The use of electronic self-report and education further enhanced the method. Not only did it save data entry time but it also provided customized, immediate patient coaching for the problems of highest intensity or distress, and a quick-to-view summary for the clinicians.

Our findings may be limited by the fact that the audio-recording was made at only one visit, a cross-section of the entire cancer treatment experience. Clinicians may have taken actions relevant to SxQOL issues after the visit or even in the next weekly visit that impacted the symptom distress outcome. Also, while we observed a significantly younger mean age of patients in the intervention group [17] who may have been more accustomed to web-based instruction than the older control group, our analyses suggest that younger age was not a significant covariate influencing the outcomes. Our sample was predominately a group of educated health care recipients being cared for in two comprehensive cancer centers; [17] thus these findings only can be generalized beyond such a sample and setting with caution.

These analyses clearly suggest that communication from patients to clinicians with regard to SxQOL may be improved with our intervention, yet many issues remain to be addressed: 1) whether the effect of the intervention was related to how often patients utilized the self-monitoring and teaching components; and 2) whether patients in the intervention group adhered more often to SxQOL management recommendations made by clinicians. Future analyses are clearly warranted to address these issues and understand more fully the effect of such intervention on patient-reported outcomes.

## Conclusions

Electronic education and coaching provided to patients with a variety of cancers of all stages resulted in significantly more specific verbal reports of SxQOL concerns made to treating clinicians in face to face visits. While there is evidence that the coached approach to describing SxQOL was adopted, the specific concerns verbalized by patients in one visit did not mediate the overall study outcome of symptom distress. The rate at which clinicians responded verbally with actions to address the concerns was not significantly different between groups. Other unknown or unanalyzed variables may explain why patients in the intervention group of the ESRA-C II trial reported lower symptom distress over the course of cancer treatment.

## Abbreviations

ESRA-C: Electronic self report assessment for cancer; SxQOL: Symptoms and quality of life.

## Competing interests

The authors declare that they have no competing interests.

## Authors' contributions

DLB conceived of the study, participated in its design and coordination, and drafted the manuscript. FH performed the statistical analysis and drafted the analytic methods and results. BH participated in study design and directed data collection for the parent study, and EF led the audio coding team. SW and RF facilitated study implementation in Seattle. All other authors participated in study design. All authors read and approved the final manuscript.

## Pre-publication history

The pre-publication history for this paper can be accessed here:

http://www.biomedcentral.com/1471-2407/14/513/prepub
